# Analysis Model of Human Resource Cross-Media Fusion Based on Deep Neural Network

**DOI:** 10.1155/2022/6069589

**Published:** 2022-06-10

**Authors:** Shengqing Ma, Shanwen Xuan, Yinjing Liang

**Affiliations:** ^1^School of Economics and Management, Qinghai Normal University, Xining, Qinghai 810000, China; ^2^School of Economics and Management, North University of China, Taiyuan, Shanxi 030051, China; ^3^School of Marxism, Taiyuan University of Technology, Taiyuan, Shanxi 030600, China

## Abstract

With the continuous deepening of enterprise system reform and the rapid development of national economy, enterprises are facing the great challenge of market competition. In the new market and social environment, the role of human resource management in enterprises becomes particularly important. To further improve the level of enterprise human resources strategic management has become an urgent problem to be solved. In the process of human resource management, enterprises are faced with complex and changeable environment and other influencing factors. Therefore, in the human resource information retrieval, this paper uses the method of deep learning to screen human resource management indicators and constructs the human resource management index system of power supply enterprises. In this paper, the nonlinear characteristics of neural network are used to establish a deep neural network human resource cross-media fusion model, which provides an operational method for enterprise human resource management. The human resource allocation relationship of enterprises is predicted, and the influencing factors and trends of personnel post-matching are analyzed. The demand forecasting results show that the neural network depth has a good fit with the enterprise staff, and the actual forecasting error is less than 3.0. It can accurately predict the human resource allocation of enterprises, improve the scientificity and effectiveness of human resource strategic decision-making, and make enterprises better adapt to the requirements of market economy. This will be of practical significance to the modernization of enterprise management.

## 1. Introduction

Human resource management has experienced unprecedented scientific and technological progress, so that human resource management has been promoted to a strategic height, human resource strategy theory, and practice research rapid development [1]. The core of enterprise competition is the competition of human resources, and enterprise internal and external environment makes the competition more intense. Enterprises should pay more attention to human resources and make reasonable planning for human resources. The core competitive advantages of enterprises make them invincible in the market competition and promote the realization of enterprise goals and strategies. Human resource strategy plays a supporting role in the realization of enterprise development strategy. With the development of economy, human resource strategy is highly valued by the majority of scholars and enterprise managers. The complex and changeable environment makes people gradually understand that all the competition of social and economic activities has obviously become the competition of human resources. Only highly competitive talents can provide support for the realization of strategic goals of enterprises, enable enterprises to gain competitive advantages in the complex and changeable competitive environment, promote enterprise development, and maximize enterprise profits. In organizational behavior research, person-job matching constitutes a new perspective to understand how organizations operate. The degree of personnel and postmatching directly affects the reasonable utilization of other resources and the overall allocation benefit of the enterprise, and it is the key factor to determine whether the enterprise can continue stable and rapid development. Therefore, now more and more human resource information systems begin to pay attention to the integration of decision support system, study how to further mine the personnel data in the enterprise human resource management system, study the relationship between internal human resources and postdemand allocation, and solve the problems existing in the effective allocation of human resources. Scientific and reasonable comprehensive evaluation determines the best position and personnel allocation to provide decision-making support, in order to improve the quality and efficiency of decision-making.

With the development of the Internet and mobile communication technology, the rise of new media such as websites, micro-blogs, WeChat, and clients competes with traditional media such as TV. As a result, the market share and advertising revenue of TV media are eroded, and the economic benefits continue to decline. To promote media convergence, it is necessary to adapt to the new situation, reform, and development in human resource management [2]. Incentive mechanism plays an important role in human resource management. Under the background of the rapid development of media integration in China, many new media and traditional media have established human resource incentive mechanisms to adapt to the development under the guidance of new ideas and management theories, which has improved the enthusiasm of employees. Effective human resources have played a positive role in improving the competitiveness of enterprises. Transmedia focuses on a cooperative communication mode adopted by media (including print media, three-dimensional media, and network media) that rely on different communication media and have different structural attributes. In order to realize complementary advantages, resource integration, and coordinated development, and expand scale effect and improve market coverage, transmedia focuses on a cooperative communication mode adopted by media (including print media, three-dimensional media, and network media). The core of transmedia lies in the “cooperative” communication between different media through “straddling” combination. Network new media also needs to make use of the content resources, human resources, policy resources, and brand influence of traditional media. The simple media integration is the connection between the media, which does not solve the practical problems of how to effectively coordinate the advantages of different media resources, how to ensure the institutionalization, standardization, and standardization of cooperation between the media, how to distribute the interests of cooperative media fairly and reasonably, and how to integrate the organizational culture of different media. Human resource strategy attaches more importance to the strategic position of human resources in the enterprise, makes the enterprise's human resource strategic decision-making more scientific and effective, improves the level of human resource management, can make full use of human resources, and can achieve the strategic goals of the enterprise.

## 2. Related Work

With the introduction of human resource management theory, the application of human resource management theory in enterprises is more extensive, and the influence of human resource management theory is gradually expanding. The research on human resources in China started from the practical staff first, and then the research on the decision-making system in research institutions, which to some extent promoted the development of human resource management research to a deeper level. In terms of HRM ideology and HRM mode, Chen L. et al. conducted in-depth analysis and discussion on the formulation and selection of HRM strategies, sorted out the current researches in this field of HRM, and proposed problems in theoretical construction, selection, and measurement of research objects [3]. Zhou et al. conducted in-depth analysis and interpretation of human resource decision-making methods from scientific and empirical perspectives, and proposed comprehensive evaluation method, index evaluation method, linear programming method, fuzzy decision-making method, and utility theory decision-making method [4]. Peng and Qi put forward the talent planning model for fuzzy integer linear programming, this model is simple, scientific, and feasible, and can reduce the enterprise cost, for the enterprise provides an effective and convenient quantitative decision method, we evaluate method applied to the human resources strategy, and through calculation we use extension evaluation to choose the best decision [5]. Hu et al. proposed the quantitative strategic planning matrix method (QSPM) for human resource strategic decision-making, which is an important analysis tool in the stage of strategic decision-making, and can objectively determine the optimal strategy and evaluate the strategy through the past analysis results [6]. Based on the dynamic capability theory, Tembhurne and Diwan studied the evolution of human resource strategic control and analyzed the strategic decision-making of human resource management in a dynamic environment, which can help enterprises effectively achieve their own goals and improve their competitiveness [7]. The rapid rise of artificial neural network theory and application research has greatly promoted the method research in various fields.

With the unprecedented development and expansion of electronic technology and computer science, the wide application of artificial neural network has become possible. Muhammad et al. applied artificial neural network to the enterprise bankruptcy early warning mechanism, proposed a fully interconnected artificial neural network structure, and applied it to the enterprise bankruptcy early warning mechanism. Compared with the traditional enterprise failure early warning method, artificial neural network can better predict the enterprise bankruptcy phenomenon [8]. Wang et al. applied artificial neural network to the calculation of the relationship between market positioning and market performance, providing a relatively scientific and objective basis for managers to make decisions and expanding the application field of neural network model [9]. Tamil Priya and Divya used BP neural network to discuss the man-post matching theory and the construction of evaluation index system, and constructed the man-post matching evaluation model, which provided a good basis for solving the management decision of man-post matching evaluation [10]. Scholars believe that the application of artificial neural network in human resource mainly focuses on human resource matching, performance evaluation, human resource demand prediction, and quantitative analysis of human resource value, but there is still a blank in the research field of human resource strategic decision-making method. In this work, artificial neural network is introduced into the human resources strategy of enterprises, and human resources and cross media are integrated. It provides a reasonable and operable tool for enterprises to choose human resources strategy, so as to improve the level of human resources management of enterprises and promote the realization of enterprise strategic goals.

## 3. Analysis Model of Human Resource Cross-Media Fusion Based on Deep Neural Network

### 3.1. Human Resource Management Index System

There are two types of human resources influencing strategic decision: internal factors and external factors. The internal influencing factors, namely, human resources' own status, can be divided into two secondary indicators, human resources' own team status and human resources' life and management status [11]. The external influencing factors, namely, the external environment of human resources, can be divided into three secondary indicators: micro, medium, and macro environment. According to the practice and data analysis of human resource strategic decision-making, 9 indicators of human resource team and 14 indicators of environment are obtained. However, after subjective evaluation, career development indicators, organizational structure indicators, and informatization development-level indicators are considered to be relatively low in importance, so they are deleted. The remaining 7 human resource team status indicators and 12 environmental indicators constitute the primary set of indicators for corporate human resource strategic decision-making, as shown in Table 1.

Human resource strategy plays a role in promoting enterprise performance. Rational allocation of human resources will maximize the potential of employees, mobilize their enthusiasm, and enhance their ability, so as to improve employee performance and labor productivity. Reduce the cost of human resources and maximize corporate profits. In order to make the screening index more scientific and reasonable, and enhance its operability and integrity, this paper uses the basic principles of modern comprehensive evaluation, on the basis of comparative analysis of the screening principles of primary indicators, and summarizes the evaluation dimensions of four primary indicators' screening; then, the suitability analysis conclusion is provided for the specific method applied to each evaluation factor to lay the foundation for integrated screening. A large number of corporate human resource strategic decision-making documents show that the principles of index selection mainly include the principle of purpose, the principle of comprehensiveness, the principle of operability, the principle of independence, the principle of representativeness, the principle of sensitivity, the principle of scientific, the principle of systematization, and so on. After analysis and arrangement, this paper refined the above index screening principles into four evaluation dimensions, as shown in Table 2.

### 3.2. Deep Neural Network Transmedia Fusion Model

In this paper, combined with the cross-media fusion analysis model constructed by deep neural network, the structure design adopts multiple structures composed of input, output, and hidden layers. The number of nodes in the input and output layers can be directly determined according to the nature of the problem to be solved. Each node in the hidden layer is connected to each node in the input layer and output layer, respectively, while the number of nodes in the hidden layer needs to be optimized based on experience or multiple trial calculations [12]. The system consists of two branches, each of which contains an input layer, several hidden layers, and a linear loss layer and a nonlinear loss layer, which are responsible for learning the potential representation of images and texts, respectively. Try to find a mapping method to maximize the fusion analysis between text and image. In the same way as the architecture, the right-most line layer of the upper and lower subnets is used to analyze the potential representation of images and text views.

The learning process of the network consists of the forward calculation process and the error backpropagation process, and the forward calculation process is as follows: the output of node *i* of the input layer is *i* = 1, 2, 3, 4, which is equal to the *i* = 1, 2, 3, 4 selected by the human resource optimization collocation model in this paper. The input *X*_*i*_ is the total output value, economic benefit, total number of employees, and proportion of technical personnel of the enterprise, respectively.

Input of hidden layer node *j*:

(1) $$\begin{array}{c} {\text{net}}_{j}=\sum_{i}X_{i}V_{ij}, \end{array}$$

(2) $$\begin{array}{c} f\left({\text{net}}_{j}\right)=\frac{1+e^{ij}}{1+e^{-ij}}. \end{array}$$

In equation (2), *V*_*ij*_ is the connection weight between input layer node I and hidden layer node *J*, whose initial value is random. *F*(*y*) is a nonlinear sigmoid transfer function [13]. Input of output layer node *j*:

(3) $$\begin{array}{c} {\text{net}}_{j}=\sum_{i}O_{i}W_{ij}, \end{array}$$

(4) $$\begin{array}{c} f\left({\text{net}}_{j}\right)=\frac{1}{1-e^{-2j}}. \end{array}$$

In equation (3), *W*_*ij*_ is the connection weight between node I of hidden layer and node *J* of output layer. Its initial value is random. Take a sample (*X*_*p*_, *Y*_*p*_) from the training sample set, input *X*_*p*_ into the network, and calculate the corresponding actual output *O*2*p*. The *O*2*p* of the optimal allocation model of human resources in this paper is the probability of technical personnel turnover [14]. The error backpropagation process is as follows: calculate the difference between the actual output *O*2*p* and the corresponding ideal output *Y*_*p*_, and adjust the weight matrix *V* and *W* by minimizing the error. The error measure of the network with respect to the PTH sample is

(5) $$\begin{array}{c} E_{p}=\frac{1}{2}\sum_{i=1}\left(Y_{p}-O_{t}\right). \end{array}$$

The error measure of the network with respect to the whole sample set is

(6) $$\begin{array}{c} E_{p}=\sum_{p}E_{p}. \end{array}$$

The network structure design needs to train the network model and adjust the weights of inter-layer nodes according to the sample data. The deep neural network algorithm adjusts the learning rate according to the local error in the training process so as to achieve the stability of the algorithm. Through the learning of a large number of training samples, the system adaptively obtains the highly nonmapping relationship between input and output [15]. The trained function of learning rate variable momentum deep network algorithm training function in MATLAB neural network toolbox will be used for training samples.

**Figure 1:**
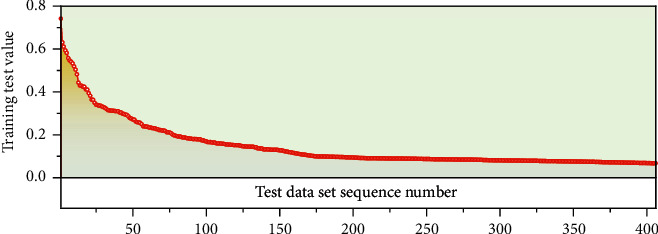
Neural network training error display diagram.

In this paper, normalized index data are used as the training input data of the deep neural network, the corresponding expected value output is the actual evaluation score value of experts, and the data of the last cycle are used as the network detection data to detect the training situation of the network [16]. The training process of the network is shown in Figure 1. During the network training, the network convergence tends to be stable, indicating that the performance is up to standard, so the training ends.

### 3.3. Human Resource Cross-Media Fusion Model Based on Deep Neural Network

**Figure 2:**
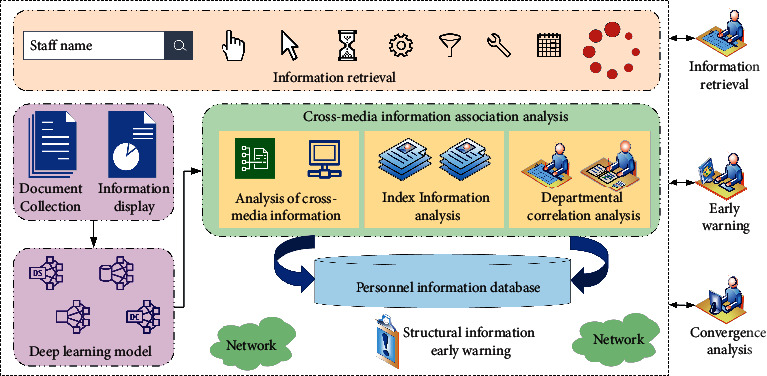
Frame diagram of human resource cross-media convergence based on deep neural network.

In the context of big data, the intelligent processing technology of transmedia information simplifies the massive, heterogeneous, multisource, and large-scale multisource transmedia intelligence data to meet the urgent demand for intelligent processing of massive transmedia intelligence data [17]. Mechanism layer is mainly to summarize the human mechanism of key cognitive processes such as attention mechanism and memory mechanism, and summarize them into an explicable, evidence-based, and realizable computing mechanism, providing support for the proposed deep neural network. Model algorithm layer integrates visual attention and memory mechanism to extract task-oriented features. The input information learns the low-level features through the convolutional neural network, and the visual attention mechanism sets the weight of each feature to obtain the global features. Finally, the memory module is used to update the features and add or update the memories at the same time. The resulting features can be used as input for the next step, which can be determined by the specific task. Through the deep neural network, the key target objects contained in the data set can be identified, and the intrinsic and valuable associations or correlations of massive multisource intelligence data can be mined and discovered, so as to accurately and quickly retrieve and extract to meet the needs of human resources. And the behavior rules of the target are further excavated to predict its human resource structure, for early warning, and to accurately understand the risk of human resource management [18]. Thus, it can effectively serve the real-time monitoring and early warning of enterprises.  Figure 2 shows the analysis framework of human resources cross-media fusion based on deep cognitive neural network in cyberspace proposed in this paper.

From the perspective of data acquisition, the personnel information studied in this paper is mainly through big data of cross-media human resource management. This framework adopts deep neural network based on visual cognitive computing and GPU acceleration method to improve learning efficiency and speed, so as to discover valuable information efficiently and accurately. Through the adoption of big data processing technology mechanisms such as model training and autonomous learning in the framework of deep learning, intelligent analysis and processing such as data association, data mining, automatic fusion matching, and association of all kinds of data such as images, texts, and videos are carried out to obtain the implicit association analysis. Through target detection and recognition, association analysis, and other methods and technologies, it can accurately and quickly retrieve and extract to meet the needs. On this basis, combined with the space-time characteristics of target data and process data sequence, the behavior law of target is further studied by using the machine learning method. The visualization technology and tools are used to visualize the analysis results, support the personnel to form insight and accurately identify the personnel structure of the enterprise, and improve the effectiveness and practicality of the analysis.

### 3.4. Human Resource Management Information Retrieval

**Figure 3:**
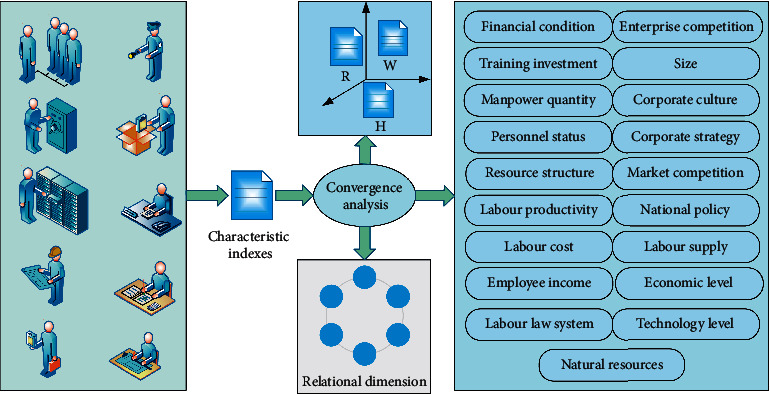
Cross-media deep learning implementation process design diagram.

Cross-media retrieval algorithm is the core of cross-media retrieval system. This paper mainly adopts cross-media deep learning to achieve its process design, as shown in Figure 3. Because the data set of multimedia data is generally unstructured or semi-structured, there is a lot of noise. Therefore, it is necessary to preprocess cross-media data for feature extraction of different media data. Read features of multimedia data from HDFS, use parallel correlation analysis method to learn potential cross-media correlation, and output model parameters and CCA spatial features [19]. Training semantic mapping for CCA spatial feature set, output classifier model and semantic space feature to HDFS. The cross-media correlation learning is implemented by parallel correlation analysis method, the LDA model is used to extract topic and topic-word distribution and the training of semantic classifier is carried out by the method proposed in this paper.

In the cross-media retrieval system, users can intuitively feel the performance of the cross-media retrieval system and whether the query is accurate through the interaction design of the system, and they submit the information of an employee to the system for query [20]. This paper designs a cross-media retrieval system for mutual retrieval of data. When the user submits the employee information query, the system preprocesses the query text and extracts the features. It uses cross-media deep learning to get the correlation matrix in the training set to represent the maximum spatial feature of the text in its correlation temper. The semantic features of the classifier in semantic space vector are trained by semantic mapping, and the semantic concept of the classifier is obtained. The cross-media retrieval algorithm is used to calculate the similarity of each image in the test set and its semantic space, the similar results are sorted and returned, and the images corresponding to the first few results in the returned results are displayed.

## 4. Results Analysis and Model Evaluation

### 4.1. Evaluate the Fitting Results

**Figure 4:**
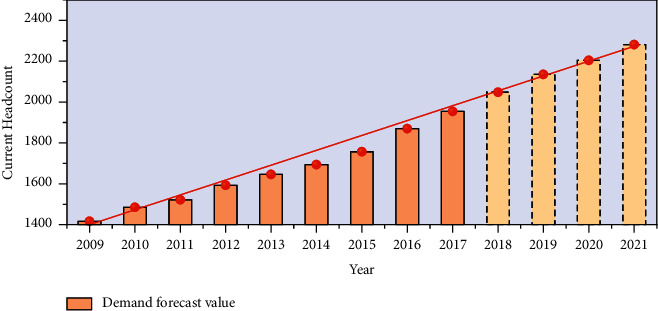
Fuzzy inference model of social security fund audit.

In order to make the analysis of the whole result more accurate and distinct, the model fitting graph can be obtained by processing the predicted result data first, and the fitting graph shown in Figure 4 can be obtained. When the user submits the personnel information query system based on the information preprocessing and feature extraction, the cross-media learning depth in the training set is used to obtain the information of the correlation matrix generated in its correlation state in the maximum spatial feature, and then the semantic features of the classifier are semantically mapped in the semantic space vector to obtain the semantic concept of the classifier. Then, the cross-media retrieval algorithm is used to calculate the similarity of each text document in the test set and its semantic space, the similar results are sorted and returned, and the corresponding text documents of the first few results in the returned results are displayed.

It can be clearly seen from the figure that there is a good fit between the predicted demand value of deep neural network and the actual number of employees of the enterprise. The demand forecasting model in the form of fusion analysis can make the demand forecasting more accurate. The predicted demand values of the number of employees from 2018 to 2021 are 2098, 2035, 2234, and 2341, respectively. From the analysis of the predicted value, we can see that the enterprise has entered a relatively stable development period since the beginning of 2000, and the demand for labor quantity has decreased to some extent, which may be due to the improvement of the enterprise's work efficiency due to technical reasons, while the demand for personnel has decreased.

### 4.2. Model Matrix Evaluation

**Figure 5:**
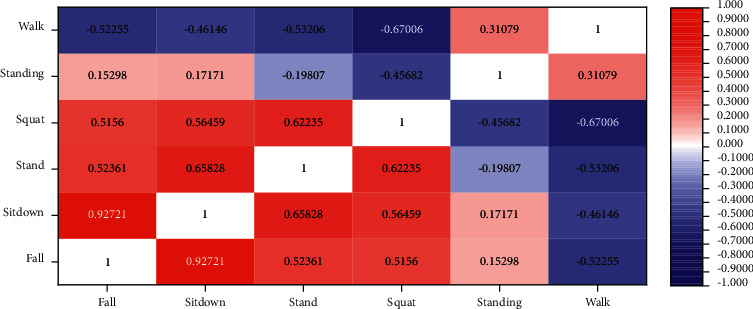
Matrix result diagram of the neural network fusion model.

Obfuscation matrix is a method to evaluate the classification performance of models by presenting the classification accuracy of models in the form of matrix. In the confusion matrix, each column represents the category result of the predicted result, each row represents the real category of the behavior, and the total number of rows and columns is equal to the amount of data of this class. The matrix reflects the normalized processing of the number in each action category, so as to evaluate the classification output performance of the network. The matrix results of the neural network fusion model are shown in Figure 5.

In the matrix graph, the diagonal element represents the probability that the predicted result is equal to the real result, while the nondiagonal element represents the action element that is not correctly predicted by the classifier. The higher the diagonal value of the confusion matrix is, the higher the accuracy of the predicted result will be. As you can see from the figure, the models for easily confused behavioral actions are 4.0% probability of being identified as sit down and squat, 3.0% probability of being identified as standing, and 3.0% probability of being identified as standing. Although there are still some classification errors, the fusion model in this paper has reduced the probability of easily confused behavior and action recognition to a minimum. Therefore, the fusion model presented in this paper has a good performance in identifying confusable actions.

### 4.3. Human Resource Structure Forecasting

**Figure 6:**
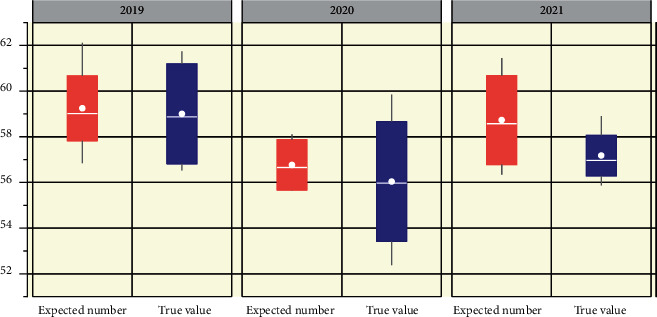
The forecast results of enterprise human resource structure from 2011 to 2021.

Taking a certain company as an example, this paper analyzes the human resource structure and its influencing factors in the past decade based on the existing data and finds that the gross output value, total benefit, total number of employees, and proportion of staff turnover of the enterprise are closely related to the human resource structure [21]. Therefore, the prediction function of human resource structure is regarded as a nonlinear function of total output value, total benefit, total number of employees, and proportion of personnel loss, and the artificial neural network is used to establish the nonlinear function, so as to avoid the complicated process of mathematical construction. Taking the total output value, economic benefit, total number of employees, the proportion of turnover and total number of employees as the input data, and the proportion of managerial personnel and scientific and technical personnel as the output data, the neural network model of enterprise human resource structure prediction is constructed. In the application process, the forecasting method shows the concern about the influence of technology on enterprise development. This forecasting method is not flexible like other quantitative forecasting methods, so it should be applied to the top-down forecasting for enterprises to enhance the flexibility of forecasting. The prediction method needs to obtain more accurate data before the prediction as a sample for training. The data from 2019 to 2021 are used as the training data to perform forward calculation first and then carry out the reverse propagation of errors and train the neural network until the errors meet the requirements. At this point, the total output value, economic benefit, demission ratio, and total number of employees of the enterprise from 2019 to 2021 are substituted into the formula to calculate, and the predicted value of the human resource structure of the enterprise from 2019 to 2021 is shown in Figure 6.

As can be seen from the forecast and analysis of the employment quantity demand of the enterprise in the figure, the enterprise belongs to the scale industry with a large number of employees, and the demand prediction method using neural network can also accurately predict the number of employment that the enterprise may need in the next few years. The model therefore predicts the proportion of managerial and technical personnel in a company from 2019 to 2021. The results show that the prediction errors are less than 3.0 and meet the requirement of prediction accuracy. From the data analysis, science and technology personnel proportion tends to rationalize, in line with the requirements of the company; the proportion of management personnel decreases year by year, while the management efficiency increases year by year. The micro-forecast of any enterprise is inseparable from the influence of macroeconomic trends which are restricted by the market environment. Therefore, in order to make the prediction of human resource structure more realistic and practical, the change and trend of the national industrial policy of the industry in the forecast period and the influence of the current international situation on the unit should also be considered in the future.

### 4.4. Human Resource Management Risk Warning

**Figure 7:**
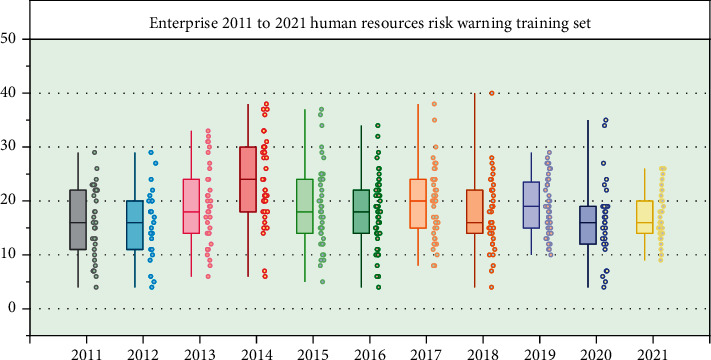
Risk output value and expert evaluation score value are not displayed as test results.

Human resource management risk early warning refers to as much as possible to avoid the happening of the risk, use some techniques and methods in advance, predict its change trend, by comparing with the target expectation deviation, identify the human resources management activities of the risk and early warning for seeking optimal controls management activities [22]. Because different risk levels have specific score ranges, different output values correspond to different risk levels; therefore, the network output results can clearly show its risk levels, and timely and accurate warning signals can be sent. In this paper, after a cycle of normalization, the index data will be used as the network test data set, the input data, and the corresponding expert evaluation value as the expected output; in comparison, it is the actual output and desired output risk test results show that the output value and the expert evaluation score are very anastomosis, as shown in Figure 7.

By the graph, you can see that human resource management risk early warning model of neural network can be used in human resource management risk early warning, the whole situation of enterprise human resources management is healthy, and the risk early warning model has good intelligence and practicability. The overall risk of human resource management can be predicted and monitored at any time. Through the example analysis, the feasibility of the model designed in this paper in predicting human resource management risks is proved. This model is established on the basis of summarizing the general human resource management risks, and its early warning function has universal applicability. When it is applicable to specific industries and enterprises, it only needs to set some risk incentives according to the specific situation and work out appropriate input layer nodes, input data, and output range. The specific function setting can be set by referring to the setting in this paper. Therefore, this early warning model has wide application.

## 5. Conclusion

With the rapid development of economic globalization and information technology, enterprises are facing more severe competition pressure. In order to adapt to the modern market demand, enterprises must optimize the allocation of human resources, and human resource management activities will have a continuous and important impact. There are many methods for forecasting and analyzing human resource demand. In the prediction analysis of human resource demand, the enterprise should consider the complex and changeable factors, the deep learning method is used to screen the human resource management indicators and build the enterprise human resource management index system, and the rationality and operability of the model have been verified. The human resource management model of neural network has strong adaptability, learning ability, and fault tolerance ability, and the prediction function of human resource structure is regarded as a nonlinear function of total output value, total benefit, total number of employees, and proportion of staff turnover. The nonlinear function model established by neural network can minimize the probability of identifying easily confused behaviors and actions, and the prediction errors of employment are all less than 3.0, which meets the requirement of prediction accuracy. The algorithm can make the weight converge to a certain value, but it cannot guarantee that it is the global minimum of the error plane. The network often has great redundancy, which also increases the burden of network learning to a certain extent. Therefore, the application of neural network in the field of human resource demand prediction still needs to be further improved and perfected according to the actual situation of enterprises. Insufficient samples and local information damage only weaken the operation of neural network moderately, but have little influence on the global effect. However, in practical application, the model may fail to accurately output the analysis results. In the later stage, more sample data are added to perform multiple training comparisons on the neural network, so as to improve the adaptability of the whole network model and improve the accuracy of analysis.

## Figures and Tables

**Table 1:** Index set of corporate human resource strategic decision-making.

Element	Serial number	Index	Importance assignment	Importance degree
Personnel situation	*X*_1_	Manpower quantity	2	0.0345
	*X*_2_	Personnel status	4	0.0690
	*X*_3_	Resource structure	3	0.0517
	*X*_4_	Labor productivity	4	0.0690
	*X*_5_	Labor cost	2	0.0345
	*X*_6_	Employee income	5	0.0862
	*X*_7_	Training investment	3	0.0517

Environmental aspect	*X*_8_	Financial condition	4	0.0690
	*X*_9_	Enterprise competition	1.5	0.0259
	*X*_10_	Size	1	0.0172
	*X*_11_	Corporate culture	1.5	0.0259
	*X*_12_	Corporate strategy	5	0.0862
	*X*_13_	Market competition	2	0.0345
	*X*_14_	National policy	4	0.0690
	*X*_15_	Labor supply	2	0.0345
	*X*_16_	Economic level	4	0.0690
	*X*_17_	Labor law system	2	0.0345
	*X*_18_	Technology level	2	0.0345
	*X*_19_	Natural resources	2	0.0345

**Table 2:** Selection principles of evaluation dimensions.

Evaluation dimensions	Selection principle
Importance *W*	Purpose; comprehensiveness; representativeness
Sensitivity *H*	Sensitivity; distinction
Relevance *R*	Independence; relevance
Acquisition difficulty *E*	Cost; operability

The four evaluation dimensions are importance, represented by *W*. The degree of differentiation is denoted by *H*; relevance, denoted by *R*; and difficulty of obtaining, denoted by *E*. These four evaluation factors highly summarize the above principles, among which importance covers the principles of purpose, comprehensiveness, and representativeness, distinction covers the principles of sensitivity and distinction, relevance covers the principles of relevance, and difficulty of obtaining indicators covers the principles of cost and operability.

## Data Availability

The data used to support the findings of this study are available from the corresponding author upon request.
